# Standards in sync: five principles to achieve semantic interoperability for TRUE research for healthcare

**DOI:** 10.3389/fdgth.2025.1567624

**Published:** 2025-06-05

**Authors:** Rhonda Facile, Catherine Chronaki, Peter van Reusel, Rebecca Kush

**Affiliations:** ^1^ CDISC, Brussels, Belgium; ^2^ HL7 Europe, Brussels, Belgium; ^3^ Catalysis Research, Austin, TX, United States

**Keywords:** clinical research, semantic interoperability (SI), data science, standards, interoperability, EHR

## Abstract

The effective and meaningful exchange of data is pivotal for patient care, informed decision-making, and advancements in research and technology. This opinion piece explores the critical role of semantic interoperability (SI) in ensuring meaningful health data sharing across diverse systems. Emphasizing the imperative of synchronizing the use of data standards, we address the challenges posed by disparate data formats and underscore the impact on patient outcomes. International, harmonized standards are presented as a cornerstone for achieving SI, while the drawbacks of proprietary standards are examined. Case studies, including the complementary use of International Organization for Standardization (ISO), Health Level Seven Fast Healthcare Interoperability Resources (HL7 FHIR), and Clinical Data Interchange Standards Consortium (CDISC) standards, offer practical insights. We offer here five simple principles [reuse existing standards where possible, avoid mapping, implement standards at the start of a project, participate in standards development activities with standards development organizations (SDOs), and work toward harmonization of standards across SDOs] for achieving semantic meaning in support of Trustworthy, Reusable, Understandable data Elements (TRUE) research data for healthcare. We hope to provide a view to a future where standards are in sync and the proposed five principles are deployed globally to ensure the conduct of trustworthy research for the sake of improving health outcomes for all.

## Introduction: the need for semantic interoperability in healthcare

Meaningful data and trustworthy research results are the essential factors synthesized to form the evidence base for clinical practice, i.e., how best to care for patients. Unfortunately, data are frequently an afterthought rather than being clearly planned along with the development of a research protocol. The manner, context (1), and format in which a data element (e.g., blood pressure) is collected and shared can mean the difference between trustworthy and accurate results vs misleading or false results, which can translate into either beneficial or harmful advice and treatments. Semantic interoperability (SI) is defined as the ability of computer systems to exchange data, with unambiguous meaning (2) and aims to share and reuse data among organizations or systems. Such reuse depends on the precise definition and sufficient context so that the data are understood and correctly interpreted, regardless of who is involved. Conveying meaning and context requires using standardized domain concepts, contextual knowledge, and formal data representation in an unbroken chain from data origination to reuse (3). Achieving SI demands reliable, standard data “at the source” for the sake of health.

Sharing data must be more than exchanging numbers or words. The meaning of that data should be the same for the sender and the recipient. Exchanging data along with its meaning so it is understood and directly usable by both parties is essential for many purposes, including (a) creating robust useful and reliable databases; (b) interpreting whether an intervention is beneficial or harmful or no better than doing nothing for a patient; and (c) enabling the automation, decision support, and data mining that unlock the value in data and information systems (4). Current advances in artificial intelligence (AI) and machine learning (ML) extend the potential value even further.

Data standards are the foundation of SI, and they are particularly valuable when widely implemented and adopted, preferably at data generation, the point of study design, and data collection. The word “standards” is a broad term that can refer to various types of standards, including metadata standards, exchange standards, content standards, various types of models, terminologies, and ontologies. In contrast to global data standards, proprietary standards (i.e., those used within a specific region or system) have limited usefulness and are typically beneficial only to the organization that developed them. The data sharing and reuse revolution, whether in supply chains or international research, pushes beyond organizational and geographical boundaries.

Robust standards are international and are developed via a fair, impartial, open, and transparent consensus-based process that allows for consideration of comments and objections, which inherently engenders adoption. Robust standards should also be harmonized such that they dovetail (extending the interoperability) and avoid duplication (conserving precious resources to develop and maintain them).

The various types of standards from different standards development organizations (SDOs) can and should be synchronized and complement one another such that, for a given data element, the relevant standardized metadata, terminology, and associated context move, along with the data itself, from the source to the data user in a capable exchange or transport standard. Implementing an exchange standard without standard content results in a loss of meaning and, in many cases, the inability to reuse the data. Without adequate metadata (i.e., information describing the characteristics of data including structural metadata such as data format, syntax, and semantics and descriptive metadata describing data contents such as information security labels) (5), assessing data quality and completeness is challenging, and provenance may be absent. The inability to demonstrate fitness for use through appropriate assessment of relevant data quality dimensions severely diminishes the value of the data. Shared data lacking sufficient semantics, context, and metadata are often meaningless and lack reuse potential. Where such reuse includes reproducibility and research replication, the value of the original research is jeopardized (6).

The use of DREAM principles “Discoverable Data with Reproducible Results for Equivalent Entities with Accessible Attributes and Manageable Metadata” has been advocated by Craig et al. (7). To encourage the use of data standards for research, the concept of Trustworthy, Reusable, Understandable data Elements (TRUE) for research is being introduced here along with five principles. The advantages are significantly greater for the research community if the metadata used to describe data elements are standardized and complement robust “in-sync” content and exchange standards. Such metadata should also be maintained by a reputable global standards development organization (SDO).

In cases where standards are developed in silos and are overlapping and redundant, the solution is frequently to map from one to another. This can be helpful, but it is never perfect; data integrity is typically lost, which negatively impacts meaning and thus the interpretation of the results. Such practices frequently lead to redundancies, especially if proprietary maps are not shared and are subject to limited quality assurance. One example is the repeated mappings between Observational Medical Outcomes Partnership (OMOP) and Health Level Seven Fast Healthcare Interoperability Resources (HL7 FHIR). At the last count, there were over 20 such projects ongoing. In addition, when one model changes, the mappings must be done again and, in some cases, go through a lengthy review and approval process that wastes precious time and resources.

The remainder of this perspective explores standards, SI, examples, and opportunities to create and maintain standards in sync. Aligning and where possible harmonizing standards globally support a learning health system through which health and healthcare data are used for research that efficiently and effectively informs and improves clinical care. It is hoped that such a system will augment and accelerate our understanding of interventions that can result in improved health outcomes. It also offers support for achieving semantic meaning when sharing (Trustworthy, Reusable, Understandable data Elements) for research and healthcare.

## How standards can be implemented in sync

There are numerous examples of how standards can work together, synchronized with one another. Such standards can be developed by the same or different SDOs working collaboratively.

HL7 has been developing health data exchange standards since 1986, following technology trends (8). Over the years, HL7 v2, HL7 v3, and Clinical Document Architecture (CDA) have been used to varying extents by the health information technology industry. Partially because they are very different standards that are striving to achieve health data exchange, their coexistence is a hurdle for the implementation community, and maintenance is a challenge for the SDO, HL7. Application Programming Interface (API) trends and research by an internal HL7 board “Fresh Look” Task Force reached a recommendation that opened new opportunities for standards in sync. The outcome and recommendation were for HL7 to advance and promote HL7 FHIR as a free broad global solution (8, 9). HL7 FHIR has proven to be a valuable exchange and interface standard. Challenges remain despite the 80/20 Pareto principle approach taken by the HL7 FHIR management group, i.e., something cannot be accepted into the core specification unless it would be useful to and used by 80% of systems around the world. HL7 FHIR has introduced a level of agility in its continuous development, linked to the level of adoption and supported by public tools, synthetic data resources, and testing that can automate parts of the implementation and conformance testing. However, HL7 FHIR resources can still be different within different countries or across different implementation guides, which decreases the ability to achieve true SI. Unfortunately, there are related HL7 FHIR resources, and there is not yet a mature governance process to keep them in sync across implementations. In addition, the primary HL7 FHIR use case is to exchange patient health data, by patient. Research use cases must support aggregation of data across patients, and research data requirements outside of that routinely captured in healthcare may not be consistently supported by HL7 FHIR. Similarly, the exchange of data to substantiate, automate, and report processes for research and registries are today not broadly supported HL7 FHIR use cases. While the Vulcan accelerator was created to advance the use of HL7 FHIR in research, the use cases remain limited and in development. Given the healthcare focus of HL7 FHIR, research use cases potentially should focus on interactions between research and the electronic health record (EHR). These are close to the HL7 FHIR scope and could be helpful in organizations around the globe leveraging EHRs to support clinical studies and registries.

The Clinical Data Interchange Standards Consortium (CDISC), a global SDO, has focused on standards to support clinical research, including use cases for aggregation of data across patients and organizations into tables and statistical data analyses (10). Over the past 25 years, CDISC has developed a vast body of content standards with associated controlled terminology. There are foundational standards that cover the most common clinical concepts (i.e., domains such as demographics, medications, lab tests, and results) and therapeutic area standards for nearly 50 therapeutic areas representing diseases that affect billions of patients around the world. These content standards are harmonized globally and can be exchanged using the original CDISC transport standard based on XML [operational data model (ODM)] or using JavaScript Object Notation (JSON) or potentially HL7 FHIR or other transport standards (11, 12). Several regulators around the world require regulatory submissions with supporting data in CDISC standard format. The CDISC Controlled Terminology is curated and maintained by the National Institutes of Health/National Cancer Institute (NIH/NCI) Enterprise Vocabulary Services (13). This is one example of how we move toward standards in sync.

SDOs have also worked together, along with other agencies, including the US Food and Drug Administration (FDA) and the NIH/NCI, in the development of an information model, i.e., the Biomedical Research Integrated Domain Group (BRIDG) model (14). This model is a CDISC standard, an HL7 standard, and an International Standards Organization (ISO) standard, which means it has gone through the development and balloting processes required for each of these SDOs. Unfortunately, the BRIDG model is an information model without public resources to support agile implementation and adoption. However, it is maintained by the NIH/NCI and has served as a reference model to map among other models, for example, in a Common Data Model Harmonization (CDHM) project, which is currently in its third phase to support academic research networks (15, 16).

Academic research institutions that wish to participate in national or global research projects or networks may be requested to provide data using a given data model, including PCORNet, OMOP, i2b2, or Sentinel. Should the academic institution wish to participate in more than one network, they currently must be able to map their data into any or all these models, and they may have their own proprietary model as the starting point. As discussed previously, such mapping not only takes time and resources but typically results in loss of meaning or data fidelity (17). In addition, these models are continuously being updated into new versions.

During the initial stage of the Clinical Data Model Harmonization (CDMH) project, the four models used by academic research networks were mapped to the BRIDG model as a reference model. This enabled the conversion of data from one model to another, but there were significant resources involved in such an exercise, and it required even more resources to maintain the base models and update mappings each time one of them was updated. The second stage employed HL7 FHIR as another model and a final conversion of data from HL7 FHIR into CDISC SDTM for use by the FDA, and the current third phase is now investigating the possibility of providing code mapping services that will automate these conversions and to include the US Office of the National Coordinator for Health Information Technology (ONC)'s core dataset for EHRs [US Core Data for Interoperability (USCDI)] in HL7 FHIR. This third phase will require detailed harmonization among the various models at the semantic level, i.e., the data element concept per ISO 11179. This is a collaborative project across Federal agencies in the United States. Concurrently, the EU has launched a project called xShare to include the HL7 FHIR and the HL7, Integrating the Healthcare Enterprise (IHE), and ISO International Patient Summary (IPS). xShare (18) is also developing an IPS for research (IPS + R). It appears that harmonization is being adopted as an approach that may replace mapping.

A good example of harmonization is the vaccine administration standard that CDISC developed during the pandemic. Rather than mapping from one standard to another, CDISC took the approach of comparing the key elements needed for tracking the administration of vaccines, based on assessments of how various organizations were collecting this information. CDISC identified the elements from the organizations that were best suited for each and developed a harmonized standard. Specifically, core data elements from the European eHealth Network Guidelines for proof of vaccination for medical purposes were aligned with the US Centers for Disease Control (CDC) Elements, the Digital Green Certificate, and the World Health Organization (WHO) Interim Guidance for Developing a Smart Vaccine Certificate (SVC). Appropriate standards were then applied to the core elements: specifically, HL7 FHIR, CDISC, three ISO standards, International Classification of Diseases (ICD), Systematized Nomenclature of Medicine—Clinical Terms (SNOMED CT), WHODrug, and Anatomical Therapeutic Chemical (ATC) classifications (see Table 1). An implementation guide was developed and published (19).

**Table 1:** Vaccine administration v1.0 summary.

Rationale and goals	Activities	Core data elements^a^
• Use case—international travel	• Harmonize a set of 20 core elements	• Vaccination Information
• Urgent need—global COVID-19 pandemic	• Based on European eHealth Network Guidelines for proof of vaccination for medical purposes—basic interoperability elements^a^	• Disease or agent targeted
• Support emerging applications with an international data standard for interoperability of core data elements and underlying metadata related to vaccine administration	• Align with:	• Vaccine/prophylaxis
• Harmonize a set of core vaccine administration data elements	• US CDC Endorsed Data Elements	• Vaccine medicinal product
• Deliver a short readily implemented standard that leverages and maps to available and widely used data standards and terminologies	• Digital Green Certificate	• Marketing authorization holder
• No new standards	• WHO Interim Guidance for Developing a Smart Vaccine Certificate (SVC)	• Manufacturer
• Follow the endorsed governance process	• Map/point to:	• Number in a series of vaccinations/doses
	• CDISC	• Batch/lot number
	• HL7 FHIR	• Date of vaccination
	• ISO standards	• Administering center
	• ISO 8601	• Health professional identification
	• ISO 3166	• Country of vaccination
	• IDMP	• Next vaccination date
	• ICD 10/11	• Patient identification Information
	• SNOMED CT	• Person name: first and last
	• WHODrug	• Person identifier
	• ATC classification	• Sex/gender
	• Develop a CDISC vaccine administration v1.0 and mapping spreadsheet	• Date of birth
		• Certificate metadata
		• Certificate issuer
		• Certificate identifier
		• Certificate valid from
		• Certificate valid until

^a^ Source: eHealth Network, Guidelines on proof of vaccination for medical purposes - basic interoperability elements, V 2. 12 March 2021. Retrieved from https://ec.europa.eu/health/sites/health/files/ehealth/docs/vaccination-proof_interoperability-guidelines_en.pdf.

Another example of collaboration and standards in sync would be the integration profiles developed with IHE (20). These include the Retrieve Form for Data Capture (RFD) (21), which can leverage a variety of standards depending on the use case (e.g., safety surveillance, clinical research, quality reporting, outbreak reporting, or healthcare). Implementation of RFD in a project on adverse event reporting demonstrated a significant return on investment. Retrieve Protocol for Execution (RPE) is another IHE profile developed to support protocol-driven research (22).

Another example is the HL7 FHIR electronic product information (ePI) standard which started with a decision of the European Medicines Agency (EMA), the Heads of Medicines Agency (HMA), and the European Commission, to develop a common standard for electronic product information or eLabeling (23). EMA decided to adopt an agile methodology and collaborate with the Gravitate-Health project, which in turn initiated a project within the Vulcan HL7 accelerator with the vision to create an HL7 FHIR Implementation Guide that will facilitate creating digital patient and physician information leaflets (ePIs) for 80% of the medicines as an initial goal. In this effort, multiple groups including the standards community of the UNICOM project are working collaboratively with EMA (24). SDOs involved include the European Committee for Standardization (CEN), International Health Terminology Standards Development Organisation (IHTSDO), HL7, and ISO. Engagement of regulators around the world facilitates alignment with the structured product label (SPL) in the case of the FDA and the currently adopted XML schema in the case of the Japanese Pharmaceuticals and Medical Devices Agency (PMDA).

## Principles for encouraging synchronous standards

Unfortunately, all too often, standards that could save significant time in the start-up and conduct of a project while facilitating reliable and exchangeable results are not used. There are several reasons for this, including but not limited to (a) lack of awareness, (b) too many choices, (c) inadequate education on how to implement, (d) poor understanding of what constitutes a standard, and (e) lack of test data and implementation tools. Robust standards are widely adopted and tested, and they should be used as is and without modification. Offering multiple options for representing a data element is not optimal when the purpose of standardization and will compromise semantic interoperability. Based upon the prior information and examples, a set of principles has been developed for healthcare and research.

The *Five Principles and Best Practice Recommendations* to optimally leverage standards and encourage synchronous use of standards from different SDOs across the healthcare and research industry include the following:

1. Reuse terminology and standards that already exist rather than investing in new development.
2. Avoid mapping whenever possible. Mapping data from one standard to another is not straightforward. Whenever mapping occurs, assumptions are made and meaning is often lost or misrepresented.
3. Implement data standards as close to the start of a project as possible, during study design and data collection, applying the principle of “interoperability at the source.” Semantic interoperability cannot be applied after data collection or downstream from data origination.
4. Participate in standards development activities with SDOs and encourage these principles, thinking about governance and maintenance. Benefit by being aware of available standards and be part of the solution so that standards meet your requirements.
5. Work toward harmonization of standards across SDOs and promote standards in sync.

## Conclusion

To unlock the power of data, semantic meaning must be maintained for the data to be trustworthy, reusable, and understood across different contexts. This is where data standards come in, in which they provide a common language for exchanging information seamlessly between organizations, including those in different countries, with semantic meaning maintained. This paper proposes a novel approach for using existing standards that are in sync with one another, addressing semantic interoperability at the source, and reducing the need for mapping, thus facilitating the exchange of higher-quality data.

We offer here five simple principles (reuse existing stands where possible, avoid mapping, implement standards at the start of a project, participate in standards development activities with SDOs, and work toward harmonization of standards across SDOs) for achieving semantic meaning in support of TRUE research data for healthcare. We hope to provide a view to a future where standards are in sync and the proposed five principles are deployed globally to ensure the conduct of trustworthy research for the sake of improving health outcomes for all.

## Data Availability

The original contributions presented in the study are included in the article/Supplementary Material; further inquiries can be directed to the corresponding author.
